# Amino Acid-Induced Chemotaxis Plays a Key Role in the Adaptation of *Vibrio harveyi* from Seawater to the Muscle of the Host Fish

**DOI:** 10.3390/microorganisms12071292

**Published:** 2024-06-25

**Authors:** Xiaoxu Zhang, Zhe Zhang, Qingpi Yan, Ziyan Du, Lingmin Zhao, Yingxue Qin

**Affiliations:** 1 State Key Laboratory of Mariculture Breeding, Fisheries College of Jimei University, Xiamen 361021, China; zhangxiaoxu1009@163.com (X.Z.); renlao75151337@163.com (Z.Z.); yanqp@jmu.edu.cn (Q.Y.); duziyan9136@163.com (Z.D.); 200561000132@jmu.edu.cn (L.Z.); 2 Key Laboratory of Health Mariculture for the East China Sea, Ministry of Agriculture, Jimei University, Xiamen 361021, China

**Keywords:** *Vibrio harveyi*, adaptation, flagella, chemotaxis, amino acids

## Abstract

*Vibrio harveyi* is a normal flora in natural marine habitats and a significant opportunistic pathogen in marine animals. This bacterium can cause a series of lesions after infecting marine animals, in which muscle necrosis and ulcers are the most common symptoms. This study explored the adaptation mechanisms of *V. harveyi* from the seawater environment to host fish muscle environment. The comprehensive transcriptome analysis revealed dramatic changes in the transcriptome of *V. harveyi* during its adaptation to the host fish muscle environment. Based on Gene Ontology (GO) and Kyoto Encyclopedia of Genes and Genomes (KEGG) analysis, flagellar assembly, oxidative phosphorylation, bacterial chemotaxis, and two-component systems play crucial roles in *V. harveyi*’s adaptation to host fish muscle. A comparison of biological phenotypes revealed that *V. harveyi* displayed a significant increase in flagellar length, swimming, twitching, chemotaxis, adhesion, and biofilm formation after induction by host fish muscle, and its dominant amino acids, especially bacterial chemotaxis induced by host muscle, Ala and Arg. It could be speculated that the enhancement of bacterial chemotaxis induced by amino acids plays a key role in the adaptation of *V. harveyi* from seawater to the muscle of the host fish.

## 1. Introduction

*Vibrio harveyi* is widely recognized as one of the most important pathogens of marine fish and invertebrates [1,2,3,4]. Infected fish exhibit a range of lesions, such as skin ulcers, eye lesions/blindness, fin rot, and severe muscle necrosis [5,6]. Meanwhile, *V. harveyi* is a normal flora in natural marine habitats and widely distributed in the ocean; it causes disease only under certain conditions, so it is regarded as an opportunistic pathogen [7,8]. Most aquatic animal pathogens are regarded as opportunistic pathogens, suggesting that they can regulate their metabolic pathways in response to the environment and adapt from a natural environment to a host environment.

Normal flora adapting to environmental changes and transforming into pathogens have been demonstrated in a limited number of pathogenic bacteria. *Escherichia coli* is one of the important model organisms, and the mechanisms of its adaptation to the host environment and transformation from normal bacteria to pathogenic bacteria during infection have been deeply studied [9,10,11]. Enterohaemorrhagic *E. coli* exists as a commensal bacterium in the intestine of bovine and can cause severe diarrheal disease if it enters into the intestine of humans. Extraintestinal *E. coli* is a harmless commensal bacterium in the microenvironment of the human intestine and can cause invasive urinary tract infections in humans once it enters the extraintestinal urinary tract [12,13,14]. *E. coli* is thought to have a unique ability for host adaptation, growing and reproducing as a harmless symbiotic bacterium in the nutrient-rich human intestinal tract and being able to transform into a pathogen in the nutrient-poor urinary tract.

From the perspective of bacterial pathogens, the host environment is complex and dynamic, containing overlapping chemical effector gradients perturbed by nutrient influx, resident microbiota competitors, and inflammatory processes [15]. Pathogens use their unique adaptation mechanisms when faced with a changing host environment. To adapt to the host environment, the expression of *psrA* in *Vibrio cholerae* was increased by 40 times to regulate fatty acid metabolism before infection so that *V. cholerae* can be exposed to mild acidic conditions to accelerate its infection of the host [16]. *V. cholerae* can employ other temporal adaptive strategies to enhance infectivity, such as changing phenotypes to overcome oxidative stress in the host [17]. To adapt to the host’s innate immune system, *Pseudomonas aeruginosa* can lose its flagellar motility to evade association and ingestion by phagocytes, which reduces inflammasome activation and benefits the bacteria during chronic infection [18]. In addition, bacteria, such as *Vibrio traumaticus* [19], *Proteus mirabilis* [20], and *Klebsiella pneumoniae* [21], have evolved iron-regulation-associated outer membrane proteins to support their survival under iron-restricted conditions during acclimatization within the host. 

There are few reports on host adaptation of aquatic animal pathogenic bacteria, and the conditions and mechanisms of the transformation of opportunistic pathogenic bacteria from normal flora to pathogenic bacteria are still unclear. Considering the serious threat of *V. harveyi* to marine animals and the symptoms of muscle ulcers caused by this bacterial infection, this study focused on exploring the adaptation processes and mechanisms of *V. harveyi* from a seawater environment to host muscle environment to reveal the mechanisms of opportunistic pathogenic bacteria host adaption in the early stage of infection. The results provide a new perspective for preventing diseases early in bacterial infection. 

## 2. Materials and Methods

### 2.1. Bacterial Strains and Materials

*V. harveyi* strain TS-628 was isolated from diseased banded grouper (*Epinephelus awoara*) and stored in our laboratory [22]. Healthy banded groupers were purchased from the Farmer’s Market of Xiamen. The medium used in this study is Zobell 2216E medium, 2216E crude muscle medium, and 2216E muscle extract medium. The components of the 2216E medium are as follows: peptone 5 g/L, yeast extract 1 g/L, FePO4 0.01 g/L, and seawater 1 L. The 2216E crude muscle medium and 2216E muscle extract medium were prepared by the methods described below. In order to exclude the influence of crude muscle on the assays, the 2216E crude muscle medium was only used for preparing the transcriptome sequencing experimental group, and 2216E muscle extract medium was used for other experiments.

### 2.2. Preparation of Muscle Extract and 2216E Muscle Medium

The fish muscle extract was prepared according to the methods described before with some modifications [23,24]. *E. awoara* was surface-disinfected with 75% alcohol (Xilong, Guangdong, China) and dissected. Under sterile conditions, the fish’s skin was uncovered, and the back muscles were taken. The muscles were washed with sterile phosphate-buffered saline (PBS, Solarbio, Beijing, China) three times, then weighed and frozen in liquid nitrogen. Some frozen fish muscle was ground and mixed with 2216E agar medium at 1:1 to prepare 2216E crude muscle plates to culture *V. harveyi*, which were used as the experimental group for transcriptome sequencing.

Some frozen fish muscle was homogenized in pre-cooled PBS at a ratio of 1:10 (*w*/*v*). Then, the homogenate was centrifuged (5000× *g*, 4 °C) for 10 min, and the supernatant was filtered through 0.45 μm and 0.22 μm pore-size filters and stored at −80 °C. The 2216E muscle extract medium was prepared by adding an equal volume of muscle extract into the Zobell 2216E Agar medium at 2× concentration.

### 2.3. Transcriptome Sequencing and Data Analysis

#### 2.3.1. Bacterial Sample Preparation

The single colony of *V. harveyi* TS-628 was inoculated into 2216E broth (Hopebio, Qingdao, China) and cultured at 28 °C, shaking at 220 rpm/min until the log phase. An amount of 100 μL of bacterial suspension was spread on the 2216E agar and 2216E crude muscle agar plates and cultured at 28 °C overnight. Then, the bacteria were gently scraped off, collected into sterile centrifuge tubes, and stored in liquid nitrogen. Bacteria samples cultured on the 2216E plates served as the control group, and bacteria samples cultured on the 2216E muscle plates served as the experimental group (muscle group). Three independent biological replicates were performed for each group.

#### 2.3.2. Transcriptome Sequencing

Bacterial samples from three independent experiments were sent to Genedenovo Biotechnology Co., Ltd. (Guangzhou, China). Transcriptome sequencing was performed on the Illumina Novaseq 6000 platform. To ensure the reliability of the subsequent bioinformatics analysis, the original sequence data were analyzed and filtered, and the high-quality sequences were compared with the *Vibrio harveyi* reference genome ASM77011v2 (GCA_000770115.2) by Bowtie 2 [25] software (version 2.2.6). The known genes were identified, and gene expressions were calculated using RNA-Seq by Expectation–Maximization (RSEM) [26]. A variance analysis of gene expression between groups was performed using edgeR [27] software (http://bioconductor.org), and FDR and log2FC were used to screen for differential genes with FDR < 0.05 and |log2FC| > 1. Differentially expressed genes (DEGs) were subjected to Gene Ontology (GO) functional annotation and Kyoto Encyclopedia of Genes and Genomes (KEGG) pathway enrichment analysis, and the significance judgment threshold was set at *q* < 0.05.

The transcriptome data were submitted to the National Center for Biotechnology Information (NCBI). The Accession Numbers of the control group are SRR21276829, SRR21276830, and SRR21276831, and the Accession Numbers of the muscle group are SRR21276825, SRR21276833, and SRR21276834.

### 2.4. qRT-PCR

Samples for transcriptome sequencing were subjected to quantitative real-time-polymerase chain reaction (qRT-PCR) validation according to the following procedures. Total bacterial RNA was extracted using the TRIzol method. The first-strand complementary DNA (cDNA) was synthesized by the all-in-one first-strand cDNA Synthesis SuperMix Kit (TransGen Biotech, Beijing, China) according to the manufacturer’s instructions. The qRT-PCR was performed on a QuantStudio ™ 6 Flex Real-Time PCR System (ABI, Carlsbad, CA, USA). The experiments were performed according to the reaction system described in the instructions for the SYBR^®^ Green Pro Taq HS Premixed qPCR kit AG11701 (Accurate Biotechnology Co., Ltd., Changsha, China). The qRT-PCR conditions consisted of a pre-incubation at 95 °C followed by 40 amplification cycles (95 °C for 20 s, 58 °C for 20 s, and 72 °C for 20 s). 16S rRNA was used as an internal reference gene to standardize and estimate the up- or down-regulation of selected genes, and three biological replicates were set up for each sample. The relative quantification of the transcript levels of the target genes was performed using the 2^−ΔΔct^ method. The primers used for the qPCR are listed in Table 1.

### 2.5. Induction of V. harveyi by Host Fish Muscle and Its Dominant Amino Acids

According to Chen et al. [28] and Wang et al. [29], the main amino acid components and contents in the muscle of *E. awoara* were glutamate (Glu, 2.93%), aspartate (Asp, 1.96%), lysine (Lys, 1.86%), leucine (Leu, 1.68%), alanine (Ala, 1.23%), and arginine (Arg, 1.23%). According to these data, amino acid solutions with corresponding concentrations were prepared using sterile water and filtered through 0.22 μm pore-size filters. Induction experiments were carried out by the method described by Ma et al. [30] with slight modifications. The amino acid solutions were added into 2216E broth according to a certain ratio to prepare 2216E amino acid broth with the final concentrations of Glu, Asp, Lys, Leu, Ala, and Arg to be 2.93%, 1.96%, 1.86%, 1.68%, 1.23%, and 1.23%, respectively. The single colony of *V. harveyi* TS-628 was inoculated into 2216E broth and cultured until the log phase. The cells were collected by centrifugation at 5000× *g* for 2 min, washed twice with 0.85% NaCl solution, resuspended in each 2216E amino acid broth and 2216E broth to adjust the concentration to OD_600_ = 0.6. The bacteria were induced at 28 °C with shaking at 220 rpm/min for 4 h. The bacteria cultured in 2216E broth served as the control group.

### 2.6. Bacterial Growth Curves

The induced bacteria were collected and resuspended to OD_600_ = 0.2 in 2216E broth. Then, diluted 100-fold, 200 μL of the diluted bacterial culture was added to the 96-well plates, and there were eight replicates for each sample. The plates were incubated at 28 °C for 24 h, and OD_600_ was measured every hour for 24 h. The data were analyzed by GraphPad Prism (v9.0.0, GraphPad Software, San Diego, CA, USA) to draw the growth curves [31].

### 2.7. Bacterial Flagella Observation

The induction experiment was carried out as mentioned above. The bacterial flagella were observed by a transmission electron microscope (Hitachi H-7650, Tokyo, Japan) according to the previously described method [32]. The induced bacteria were collected and resuspended in PBS to a concentration of OD_600_ = 0.4. The 300-mesh copper mesh was placed in each group of bacterial suspension for 5 min. The excess samples were absorbed with filter paper, while the rest of the samples were transferred to 2% phosphotungstic acid (Solarbio, Beijing, China) for 1 min. The surface of the copper mesh was rinsed quickly with sterile water and dried. Finally, the bacterial flagella were observed under the transmission electron microscope. The flagellum lengths of at least three bacterial cells of each group were measured using Image J (version 1.54, National Institutes of Health, Bethesda, MD, USA), and the relative flagellar length = the flagellar length of the experimental group/the flagellar length of the control group.

### 2.8. Bacterial Swimming, Swarming, and Twitching

Bacterial swimming, swarming, and twitching assays were performed as previously described [33,34,35]. The induced bacteria were prepared according to the above method, collected, resuspended, and adjusted to OD_600_ = 0.2. Then, 2 μL of bacterial suspension was inoculated vertically on 2216E semisolid agar plates (0.4% agar) and incubated at 28 °C for 4 h, with the swimming colonies’ diameters measured. For the bacterial swarming assay, the induced bacteria suspension was adjusted to OD_600_ = 1. About 2 μL bacterial suspension was inoculated on the 2216E semisolid agar plates (0.6% agar) and incubated at 28 °C for 24 h. The swarming colony’s diameters were measured. For the twitching assay, 2 μL OD_600_ = 1 bacterial suspension was inoculated to the bottom of the Petri dish from the 2216E plates (1% agar). After incubation at 28 °C for 24 h, the medium was gently discarded, and the bacterial twitching area was stained with 0.1% crystal violet (Solarbio, Beijing, China). The bacterial motility zones at the agar/Petri dish interface were measured by Image J. Relative swimming/swarming diameter = the colony diameter of the experimental group/the colony diameter of the control group. Relative twitching area = the twitching area of the experimental group/the twitching area of the control group.

### 2.9. Bacterial Chemotaxis

The chemotaxis assays were carried out following previously reported methods [36]. The inducted bacteria were prepared according to the above method. Then, the induced bacteria were resuspended with sterile PBS and adjusted to OD_600_ = 0.2. About 0.4 mL of bacterial suspension was aspirated with a 1 mL syringe. An amount of 5 μL of the muscle extract was added from one end of the capillary tube, and the other end was sealed. Then, the open end of the capillary tube was dipped into the bacterial suspension and horizontally incubated at 28 °C for 1 h. The muscle extract in the capillary was blown into 995 μL 2216E medium in the eppendorf tubes, and the number of bacteria in the muscle extract was counted by plate counting. Chemotactic index = the number of chemotactic bacteria of the experimental group/the number of chemotactic bacteria of the control group.

### 2.10. Bacterial Adhesion

Adherent bacteria were observed by the method described by Wu et al. [37]. Briefly, 20 μL muscle extract drop was placed in the center of a 22 × 22 mm^2^ slide and spread evenly. After the extract on the slide was dried naturally, it was fixed with 4% methanol for 30 min. An amount of 200 μL induced bacteria resuspension (OD_600_ = 0.4) was applied to the extract area. The slides were placed in a humid environment and incubated at 28 °C for 2 h. The slides were then rinsed with sterile PBS for 3 times. The adherent bacterial cells were removed, and then the slides were air-dried. After being fixed with 4% methanol again for 30 min, 200 μL crystal violet solution (0.1%) was used to stain the slides for 3 min. The stained slides were washed with PBS, and the stained bacteria cells were observed under an optical microscope (Zeiss Axiolab 5, Oberkochen, Germany).

The relative adhesion coefficient was evaluated by the methods described previously with some modifications [38,39]. The protein concentration of muscle extract was adjusted to 0.5 mg/mL in Hepes–Hank’s buffer (Solarbio, Beijing, China). An amount of 100 μL muscle protein was added to each well of the 96-well plate and incubated at 4 °C for 24 h. After incubation, the 96-well plate was washed twice with 200 μL Hepes–Hank’s buffer to remove the unbound mucus protein. The induced bacteria were collected and resuspended in Hepes–Hank’s buffer to adjust the concentration to 1 × 10^8^ colony-forming units (CFU)/mL. Hoechst 33528 was added to the bacterial suspension to a final concentration of 2 μg/mL, and the bacteria were stained at 28 °C for 30 min under light-protected conditions. Then, the bacteria were collected and washed twice with PBS and resuspended in Hepes–Hank’s buffer. The stained bacteria (100 μL) were added to the 96-well plate prepared above and incubated at 28 °C for 1 h. After that, the 96-well plate was washed twice with Hepes–Hank’s buffer to remove unadhered bacteria, 200 μL 1% SDS-0.1 mol/L NaOH was added to each well and incubated at 60 °C for 1 h for cell lysis. An amount of 100μL of unstained bacteria served as the negative control, and 100 μL of stained bacteria without incubation served as the positive control. The fluorescence intensity was measured by a multifunctional enzyme marker (BioTek Synergy H1, Santa Clara, CA, USA), with an excitation wavelength of 340 nm and an emission wavelength of 460 nm. Adhesion rate (%) = (fluorescence intensity of experimental group − fluorescence intensity of negative control)/(fluorescence intensity of positive control − fluorescence intensity of negative control) × 100%. Relative adhesion coefficient = the adhesion rate of each group/the adhesion rate of the control group.

### 2.11. Bacterial Biofilm

Bacterial biofilm assays were performed according to the methods described by Mao et al. [40] and Wooten et al. [41]. The induced bacteria were collected and adjusted to OD_600_ = 0.2. Then, the bacterial suspension was diluted 10-fold. The bacterial suspension was added to the 96-well plates (200 μL per well), with three replicate wells for each sample. The 96-well plates were incubated at 28 °C for 36 h, and the wells were washed twice with PBS and air-dried, stained with 0.1% crystal violet for 3 min, and washed three times with PBS, and the biofilm was dissolved in 200 μL of 33% acetic acid. A multifunctional enzyme marker determined the absorbance value at an optical density of 590 nm (OD_590_ nm). The relative biofilm formation ability = OD_590_ of each group/OD_590_ of the control group. 

Meanwhile, 2 mL of diluted bacterial suspension was added to a 6-well plate with a 22 mm diameter round glass coverslip and incubated at 28 °C for 36 h. The round glass coverslips were taken out, slowly rinsed twice with 1 mL PBS to remove unadhered cells, air-dried, fixed in 4% glutaraldehyde (Yuanye, Shanghai, China) for 30 min, rinsed twice with PBS, and stained with SYBR Green I dye (Solarbio, Beijing, China) for 30 min in a light-protected environment. The slides were rinsed twice with PBS, air-dried, and observed under a confocal laser microscope (Leica TCSSP8, Wetzlar, Germany).

### 2.12. Effects of Host Muscle and Its Dominant Amino Acids on Gene Expression Relative to the Chemotactic Process of V. harveyi

The qPCR was used to evaluate the expression of methyl-accepting chemotaxis proteins (MCPs) (PctB, Tar, McpP, McpU), chemotaxis proteins (CheA, CheV, CheW, CheY), and flagellins (FliC, FliG, FliN, FlgE, MotA) after induction by host fish muscle and its dominant amino acids. The bacteria were induced according to methods described above and collected to extract RNA. The kit and reaction procedures were consistent with those mentioned above. The primers are listed in Table 1.

### 2.13. Data Analysis

The data of the experimental group in the qPCR were compared with those of the corresponding control group by *t*-tests, and *p* < 0.05 was displayed as *, *p* < 0.01 was displayed as **, and *p* < 0.001 was displayed as ***. All the data were analyzed by a one-way analysis of variance (ANOVA), followed by Duncan’s test using Statistical Package for Social Sciences (SPSS) 26.0 software (Chicago, IL, USA). The results, based on three independent biological experiments, are expressed as mean ± standard deviation (SD) with statistical significance defined as *p* < 0.05.

## 3. Results

### 3.1. Transcriptome Analysis

**Figure 1:**
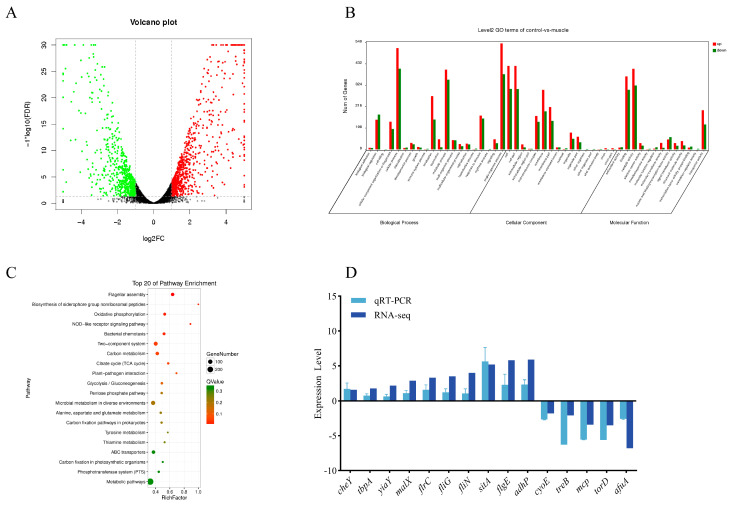
Comparative transcriptomic analysis of muscle-cultured and wild-type strains of *V. harveyi*. (**A**) Volcano map of DEGs between control and muscle. Green represents down-regulation, red represents up-regulation. (**B**) Histogram presentation of clusters of GO classification of differentially expressed genes. (**C**) KEGG enrichment analysis bubble chart. (**D**) Transcriptome sequencing verification.

A transcriptomics analysis revealed that a total of 1341 DEGs were identified in the muscle group compared to the control group, of which 725 genes were significantly up-regulated and 616 genes were significantly down-regulated (Figure 1A). The functions of these 1341 DEGs were annotated and analyzed by GO. Most DEGs were involved in the following functions: cellular processes, single-organism processes, metabolic processes, cell parts, catalytic activity, and binding (Figure 1B). A KEGG pathway enrichment analysis showed that the DEGs were enriched in the flagellar assembly, oxidative phosphorylation, bacterial chemotaxis, two-component system pathways (Figure 1C). The results of the qPCR were consistent with the trends of transcriptome sequencing, indicating the reliability of the sequencing (Figure 1D). 

### 3.2. Effects of Host Muscle and Its Dominant Amino Acids on the Flagellum of V. harveyi

**Figure 2:**
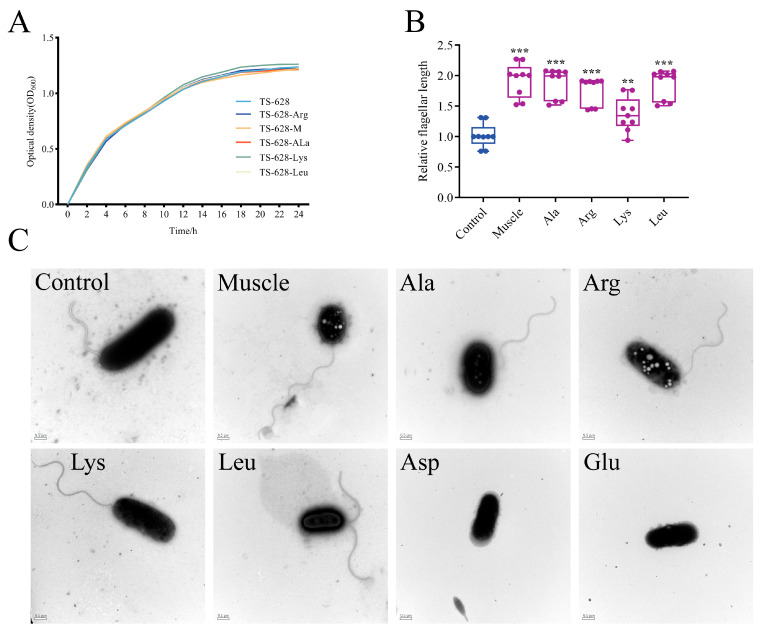
Effects of host muscle and its dominant amino acids on growth and flagella of *V. harveyi*. (**A**) Growth curves. (**B**) Relative flagellar length. (**C**) Morphology of bacterial flagella induced by muscle and its dominant amino acids. Data are represented using a boxplot graph showing the median, inter-quartile range, upper and lower quartiles, and whiskers. The circles represent the original data. ** means *p* < 0.01, *** means *p* < 0.001.

The host muscle and its dominant amino acids, except Asp and Glu, did not affect the growth of *V. harveyi* (Figure 2A). However, Asp and Glu almost inhibited bacterial growth. After detection, the pH of the 2216E medium with Asp or Glu added was only about 3.2, the bacteria did not grow after inoculation in these media, and the pH of the medium did not change. After induction, the relative flagellar length of *V. harveyi* was 1.9, which indicated that the length of the flagella was almost doubled. In addition, Ala, Leu, and Arg significantly influenced flagellar length (Figure 2B). Electron microscope observation found that the number of polar flagella of *V. harveyi* changed from one to two after induction by Leu, indicating that Leu induction affected the length of bacterial flagella and the number of flagella (Figure 2C). In contrast, *V. harveyi* lost flagella after induction by Asp and Glu.

### 3.3. Effects of Host Muscle and Its Dominant Amino Acids on Swimming, Swarming, and Twitching of V. harveyi

**Figure 3:**
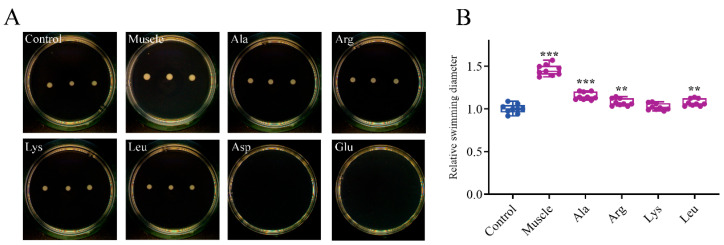
Effects of host muscle and its dominant amino acids on swimming motility of *V. harveyi*. (**A**) Bacteria swimming on semisolid plates. (**B**) Relative swimming diameter. Data are represented using a boxplot graph showing the median, inter-quartile range, upper and lower quartiles, and whiskers. The circles represent the original data. ** means *p* < 0.01, *** means *p* < 0.001.

**Figure 4:**
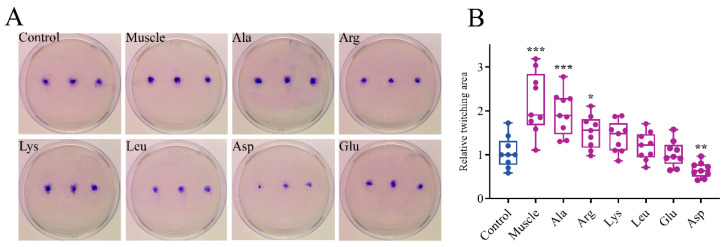
Effects of host muscle and its dominant amino acids on twitching motility of *V. harveyi*. (**A**) Crystal violet-stained twitching area. (**B**) Relative twitching area. Data are represented using a boxplot graph showing the median, inter-quartile range, upper and lower quartiles, and whiskers. The circles represent the original data. * means *p* < 0.05, ** means *p* < 0.01, *** means *p* < 0.001.

**Figure 5:**
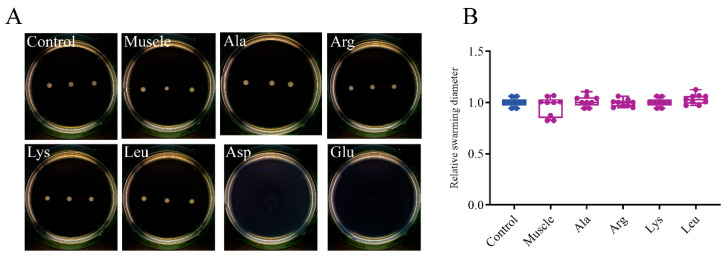
Effects of host muscle and its dominant amino acids on swarming motility of *V. harveyi*. (**A**) Bacteria swarming on semisolid plates. (**B**) Relative swarming diameter. Data are represented using a boxplot graph showing the median, inter-quartile range, upper and lower quartiles, and whiskers. The circles represent the original data.

Figure 3 displays that the relative swimming diameters of muscle-induced bacteria and Ala-induced bacteria were about 1.46 and 1.15, respectively. The relative twitching areas of muscle-induced bacteria and Ala-induced bacteria were about 2.09 and 1.96, respectively (Figure 4). The swimming and twitching ability of *V. harveyi* was significantly enhanced under the induction by muscle and Ala. In contrast, muscle and amino acids exhibited no effect on the swarming ability of *V*. *harveyi* (Figure 5).

### 3.4. Effects of Host Muscle and Dominant Amino Acids on the Chemotaxis of V. harveyi

**Figure 6:**
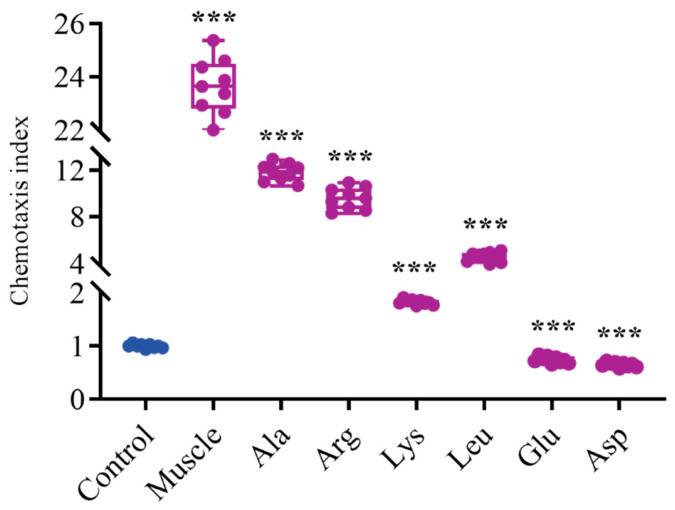
Effects of host muscle and its dominant amino acids on chemotaxis of *V. harveyi*. Data are represented using a boxplot graph showing the median, inter-quartile range, upper and lower quartiles, and whiskers. The circles represent the original data. *** means *p* < 0.001.

Results in Figure 6 show that after muscle and Ala induction, the chemotaxis ability of *V*. *harveyi* was 23.7 times and 11.8 times that of the control group, respectively. Bacterial chemotaxis ability was significantly enhanced after induction by Arg and Leu. However, the chemotaxis ability of *V. harveyi* significantly decreased under the induction of Asp and Glu.

### 3.5. Effects of Host Muscle and Its Dominant Amino Acids on the Adhesion of V. harveyi 

**Figure 7:**
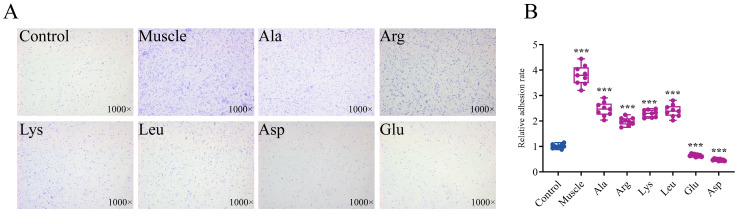
Effects of host muscle and its dominant amino acids on adhesion of *V. harveyi*. (**A**) Adhered bacteria after induction under optical microscope. (**B**) Relative adhesion rate of *V. harveyi* after induction. Data are represented using a boxplot graph showing the median, inter-quartile range, upper and lower quartiles, and whiskers. The circles represent the original data. *** means *p* < 0.001.

The results in Figure 7 show that the adhesion ability of *V. harveyi* is 3.8 times and 2.46 times that of the control after muscle and Ala induction, respectively. After Arg, Lys, and Leu induction, bacterial adhesion ability was also significantly enhanced. Meanwhile, Asp and Glu decreased the adhesion ability of *V*. *harveyi*.

### 3.6. Effects of Host Muscle and Its Dominant Amino Acids on Biofilm Formation of V. harveyi

**Figure 8:**
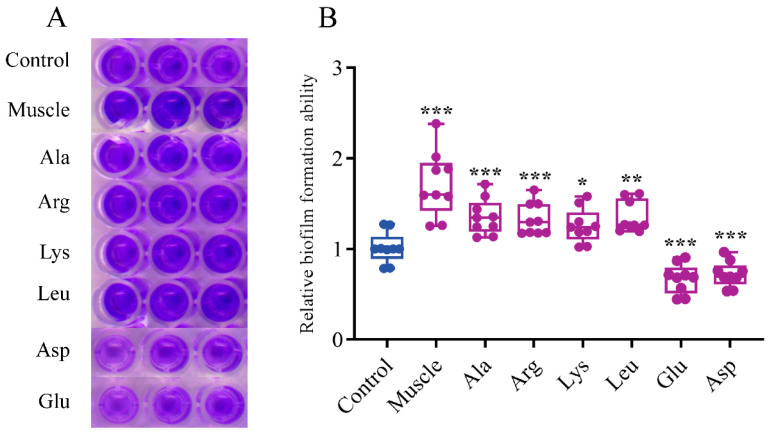
Bacterial biofilm formation was detected using the crystal violet method. (**A**) Crystal violet-stained biofilm image of *V. harveyi* after induction in a 96-well plate. (**B**) Relative biofilm formation ability of *V. harveyi* after induction. Data are represented using a boxplot graph showing the median, inter-quartile range, upper and lower quartiles, and whiskers. The circles represent the original data. * means *p* < 0.05, ** means *p* < 0.01, *** means *p* < 0.001.

**Figure 9:**
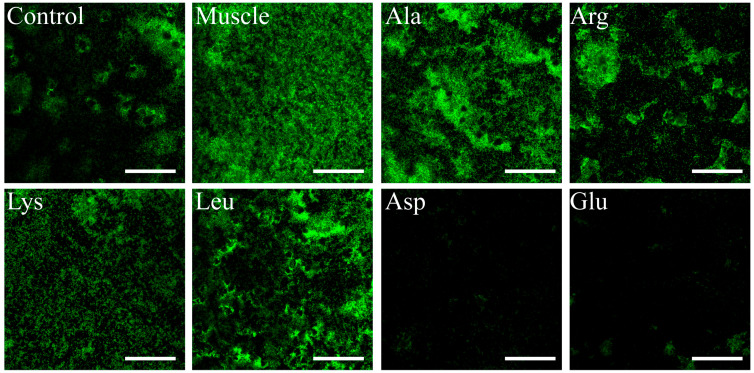
Bacterial biofilm formation was observed by a confocal laser microscope. Cells in the biofilms were stained with SYBR Green I dye; bacteria exhibited green fluorescence. Bars, 50 μm.

Both the results of the crystal violet method (Figure 8) and confocal laser microscope (Figure 9) showed that the biofilm formation ability of *V. harveyi* was significantly improved after induction by muscle and dominant amino acids (except Asp and Glu), among which muscle had the strongest ability to induce bacterial biofilm formation. However, the ability of bacterial biofilm formation decreased under Asp and Glu induction. 

### 3.7. Effects of Host Muscle and Its Dominant Amino Acids on the Expression of Genes Related to the Bacterial Chemotaxis Process 

**Figure 10:**
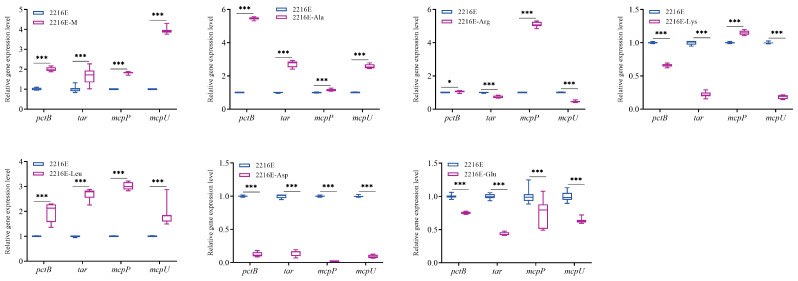
Effects of host muscle and its dominant amino acids on the expression levels of some methyl-accepting chemotaxis proteins. Data are represented using a boxplot graph showing the median, inter-quartile range, upper and lower quartiles, and whiskers. * means *p* < 0.05, *** means *p* < 0.001.

To investigate the effect of muscle and its dominant amino acids on the chemotaxis process of *V. harveyi*, the expression levels of some important methyl-accepting chemotaxis protein genes, chemotaxis genes, and different parts of flagella genes under the induction of muscle and its dominant amino acids were evaluated. And the functions of the relevant genes are listed in Table 2. Figure 10 results suggest that the expression levels of all detected MCPs were significantly up-regulated under the induction of muscle and Ala. The expression levels of some MCPs were significantly up-regulated under the induction of Arg, Lys, and Leu, and the expression levels of all detected MCPs were significantly down-regulated under the induction of Asp and Glu.

**Figure 11:**
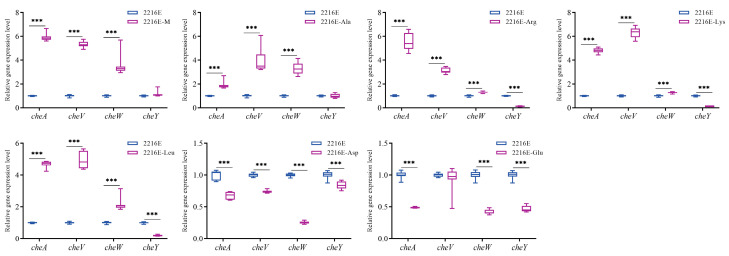
Effects of host muscle and its dominant amino acids on the expression levels of chemotaxis genes. Data are represented using a boxplot graph showing the median, inter-quartile range, upper and lower quartiles, and whiskers. *** means *p* < 0.001.

The *cheA*, *cheV*, and *cheW* expression levels were significantly up-regulated under muscle and dominant amino acid induction, except Asp and Glu. However, the expression of *cheY* remained unchanged or significantly down-regulated under induction. The expression levels of all chemotaxis genes were down-regulated under the induction of Asp and Glu (Figure 11).

**Figure 12:**
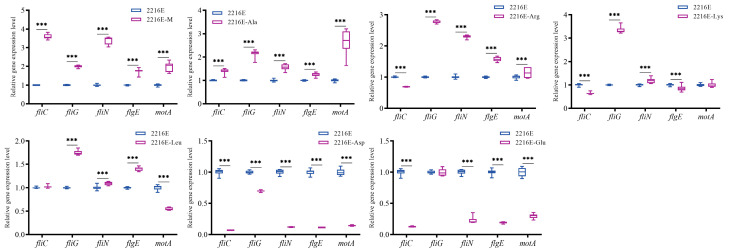
Effects of host muscle and its dominant amino acids on the expression of flagellar genes. Data are represented using a boxplot graph showing the median, inter-quartile range, upper and lower quartiles, and whiskers. *** means *p* < 0.001.

The results in Figure 12 indicate that the expression levels of detected flagellar genes were significantly up-regulated under the induction of muscle and Ala. The expression levels of some flagellar genes were significantly up-regulated under the induction of Arg, Lys, and Leu. The expression levels of almost-detected flagellar genes were significantly down-regulated under the induction of Asp and Glu.

## 4. Discussion

Microorganisms utilize various abilities to increase their fitness within the host or reduce the host’s fitness during the interaction process, and understanding bacterial host adaptability allows for a better investigation of the pathogenicity mechanisms of pathogens. This study found that the transcriptome of *V. harveyi* changed dramatically during the transition from a natural environment to a host muscle environment, in which the DEGs accounted for 23.7% of the total genes of the *V. harveyi* genome. The phenomenon of regulating metabolic pathways to make great changes to adapt to environmental changes has been reported in many microorganisms. When *Microbacterium sediminis* was exposed to low temperatures, the expression of 37.3% of its genes changed, and when *M. sediminis* was exposed to high-pressure environments, the expression levels of 35.43% of its genes significantly changed [42]. In the early stages of *Staphylococcus aureus* infection, the expression levels of several genes encoding pro-inflammatory molecules (IL8, IL1B, OSM, and CXCR1, etc.) show a dramatic increase, with the expression of IL8 increasing up to 8196-fold [43,44].

The transcriptome analysis also revealed that the pathways, such as flagellar assembly, oxidative phosphorylation, bacterial chemotaxis, and the two-component system, played central roles in the host muscle adaptation of *V. harveyi*. The flagella and chemotaxis of *V. harveyi* were crucial in its host muscle adaptation process. Some bacteria expressed two flagellar systems responsible for swimming motility and swarming motility, respectively. The single polar flagellum is responsible for the swimming motility in liquid environments; the lateral flagellum is stimulated and responsible for the swarming ability [45]. The expression of polar flagellar genes is continuous, while the lateral flagellar genes are induced by many factors [45]. But the correlation between lateral flagella and swarming is not absolute; some bacteria with flagella originating from a single cell pole can swarm [46]. In this study, muscle and amino acids exhibited effects on the swimming and twitching abilities of *V. harveyi*, but no effects on the swarming ability of *V. harveyi*, which may be related to the lack of lateral flagella. It could be speculated that polar flagella and chemotactic swimming motility enable *V. harveyi* to move purposefully and colonize the most suitable ecological niche of the host, which is conducive to bacterial optimal growth, reproduction, and further expansion.

The key role of chemotactic motility in bacterial environmental adaption has been demonstrated. Impaired chemotaxis, such as *cheY1* and *cheY2* mutations, reduced the chemotaxis of *Helicobacter pylori* to mucus proteins, preventing the pathogen from adapting the host gastric mucus environment and occupying the ecological niche in it so that the *H. pylori* was flushed out of the stomach with the flow of gastric fluid [29,47]. In the early stage of *V. cholerae* infection, chemotaxis proteins (CheY-3, CheA-2, and CheZ), and motility proteins (FlaA) are required to initiate the expression of the virulence gene (*toxT*), indicating that *V. cholerae* can migrate to the optimal location of the host intestine through chemotaxis for virulence factor expression [48]. To adapt to a nutrient-stress environment lacking fatty acids, the expression levels of chemotaxis genes (*cheA*/*Y)* of *Listeria monocytogenes* were significantly up-regulated by 28.44 and 4.14 times, respectively. The overexpression of chemotaxis genes enabled *L. monocytogenes* to promote biofilm formation, which is an adaptive mechanism for pathogens to cooperate to overcome nutrient deficiency [49]. For *V. harveyi* in this study, the host muscle is rich in amino acids, which induce bacteria to synthesize many MCPs, flagella, and chemotaxis-associated proteins as reserves to prepare for further proliferation and expansion to the optimal sites of the host. 

Our study also found that among the dominant amino acids of the host muscle, Ala has the strongest and most comprehensive induction ability, almost equivalent to muscle induction ability. After the induction of Ala, the expression of flagella and chemotaxis genes of *V. harveyi* was up-regulated, and the ability of swimming, twitching, chemotaxis, and adhesion was enhanced. Ala may be the key amino acid in inducing host muscle adaptation of *V. harveyi*. Amino acids are the fundamental components of proteins. Many bacteria depend on the amino acids of the host as the primary energy and carbon source, so the precise regulation of amino acids is of great significance for bacterial environmental adaptation [50]. Ala is one of the essential amino acids involved in protein synthesis. As we know, Ala can be metabolized to pyruvate, and pyruvate can be converted to acetyl-coenzyme A, which is the major upstream input for the TCA cycle [51]. Furthermore, Ala can transfer the amino group to α-ketoglutarate and produce pyruvate and L-glutamate, and Glu is the only amino acid that undergoes rapid oxidative deamination by glutamate dehydrogenase, which uses NAD or NADP as a coenzyme and generates NADH or NADPH [52]. And Ala can connect the metabolic pathways of carbohydrates and nitrogen. For example, Ala can affect the metabolic pathways of nearly 20 amino acids in *V. alginolyticus*, indicating that Ala is significant to bacterial metabolism [53]. It can be imaged that through these metabolic pathways, *V. harveyi* can generate a large quantity of energy to meet the needs of bacterial chemotactic movement and provide sufficient energy for bacteria to adapt to their environment, further growth and reproduction, and eventually establish infection. 

There are also studies showing that amino acid induction affected bacterial flagellar length and changed the morphology of bacterial flagella. For example, under the induction of Leu, the flagella length of *V. harveyi* increased, and the morphology of diflagella also appeared. A study on *Burkholderia cenocepacia* found that the number of flagella increased in a sputum environment rich in mucus, amino acids, and carbohydrates. In the presence of Arg, Glu, His, Phe, and Pro, the motility of *B. cenocepacia* increased by about two times, but the flagellin expression did not increase. Since bacterial chemotaxis was correlated with amino acid utilization, *B. cenocepacia* could enhance its motility by enhancing chemotaxis rather than increasing flagellin synthesis [54,55]. 

However, after induction of Asp and Glu, the most abundant amino acids in the host muscle, *V. harveyi* lost motility and could not even grow. We speculated Asp and Glu are both acid amino acids, which can change the pH of the medium. On the other hand, *V. harveyi* is a marine bacterium, which is adapted to the alkalescent seawater environment, so the bacteria cannot survive and grow well under the condition of high Asp and Glu content. The study also reported that high concentrations of Val and Leu inhibited bacterial growth [53]. These studies suggested that different bacteria have different needs and reactions to ammonia acids, and pathogenic bacteria adaption to the host is an extremely complex process. There are various induction and inhibition factors in the host environment existing simultaneously. Under the combined action of these factors, pathogenic bacteria can adapt to the host and further cause infection through timely regulation of expression and metabolism.

## 5. Conclusions

*V. harveyi* underwent drastic changes in transcriptome during its adaptation from seawater to the muscle of the host fish. The enhancement of bacterial chemotaxis induced by amino acids played a key role in this adaptation process. Alanine displayed the most comprehensive induction ability in the adaptation of *V. harveyi* to host muscle.

## Figures and Tables

**Table 1:** Primers for qRT-PCR.

Primers	Sequences
16S rRNA-F/R	5′-GGAGCAAACAGGATTAGATACCC-3′/5′-TTCTGATTCCCGAAGGCAC-3′
*cheY*-F/R	5′-CGGAAGGTGAGATGATTG-3′/5′-CTTTTCGCGTATGGATAA-3′
*tbpA*-F/R	5′-CTTAACCGCCTTCGCTTGG-3′/5′-GCTTTTTGGAGGATTCGCC-3′
*yiaY*-F/R	5′-GTCTTGCTTTGCTCCCTGTC-3′/5′-CTATCCTCACCTTGCGTTCC-3′
*malX*-F/R	5′-CTCGTCTTCGCTTGTCTGTT-3′/5′-GGCTTGCATTTCATTCTTCA-3′
*flrC*-F/R	5′-TTGTGGCATGTCATCAGAAG-3′/5′-CGAACGGACGAAGTAGGTAG-3′
*fliG*-F/R	5′-TCTGCTCGTGGTGTCATCC-3′/5′-TTCATCGCCATACAGGTTG-3′
*fliN*-F/R	5′-AAGAGCATTCCAGTGACCGTA-3′/5′-CGACTTCTGCTTGTCCCATTA-3′
*sitA*-F/R	5′-TGCTGAAGCATACAAAAAGC-3′/5′-TCACTGAATACCGCAGAGAT -3′
*flgE*-F/R	5′-TCAACTCTTCTTACACCACC-3′/5′-TCTACATTCACTTGCCATTC-3′
*adhP*-F/R	5′-GTGTTGGTGTTCCTTGGTTG-3′/5′-TGTTACTTTGAGCGCCTTAT-3′
*cyoE*-F/R	5′-ACCATGTGGTACAAACGAA-3′/5′-TAACGGGAAGAACAGGAAT-3′
*treB*-F/R	5′-ACAAAGCCGATACAAAACAG-3′/5′-TTCAGCAAGGTGGGAAATAC-3′
*mcp*-F/R	5′-TTGCTGCTGGTGACTCTGA-3′/5′-TTTCTTTTGCCGCTTCTTT-3′
*torD*-F/R	5′-GAGAAACGCGCAGAAATC-3′/5′-CCAAGACCCGCTAAAAAG-3′
*afuA*-F/R	5′-ACTCTTACCGTCAACCTTTC-3′/5′-GATTTTGCTGTCTACCTTTT-3′
*fliC*-F/R	5′-CAACGCAAACTCAGCACAA-3′/5′-TACGAACAGCCACATCCAA-3′
*motA*-F/R	5′-TGGGTTCGGTATTCTTGC-3′/5′-GTTTCGTTTCACTCGCTG-3′
*pctB*-F/R	5′-TGTGGTGTTCGTGGTGTTACTG-3′/5′-ACTCAATGTCATCACTTCGGTCAA-3′
*tar*-F/R	5′-CGGCAGCGATTGAGCAAGTAA-3′/5′-TTGAGTGCGATGAGCCAGTGTA-3′
*mcpP*-F/R	5′-CGTATTTGCGATGGGGAT-3′/5′-GGCGGACGATGATTTTTC-3′
*mcpU*-F/R	5′-GCTATCTCGCACCTTTCTTC-3′/5′-CAATCGTCTTTACGCTCACC-3′
*cheA*-F/R	5′-GCTTCCTATCATTGGCA-3′/5′-AGTGGTTCACGGTCTTG-3′
*cheV*-F/R	5′-TGGCACAAGAGGGTAGTAT-3′/5′-TCAATGAGGAGTGAAGGAT-3′
*cheW*-F/R	5′-CTACACAGAAATCGCTCC-3′/5′-CTTCACCTTCCATCAAAC-3′

**Table 2:** The functions of genes related to the chemotaxis process of *V. harveyi*.

Gene Name	Description or Predicted Function	Gene Name	Description or Predicted Function
*pctB*	methyl-accepting chemotaxis protein	*cheW*	two-component system, chemotaxis family, purine-binding chemotaxis protein CheW
*tar*	methyl-accepting chemotaxis protein II, aspartate sensor receptor	*cheY*	two-component system, chemotaxis family, chemotaxis protein CheY
*mcpP*	methyl-accepting chemotaxis protein	*fliC*	flagellin
*mcpU*	methyl-accepting chemotaxis protein	*fliG*	flagellar motor switch protein FliG
*cheA*	two-component system, chemotaxis family, sensor kinase CheA	*fliN*	flagellar motor switch protein FliN
*cheV*	two-component system, chemotaxis family, chemotaxis protein CheV	*flgE*	flagellar hook protein FlgE
		*motA*	chemotaxis protein MotA

## Data Availability

The original contributions presented in the study are included in the article, further inquiries can be directed to the corresponding author.

## References

[1] Triga A., Smyrli M., Katharios P. (2023). Pathogenic and opportunistic *Vibrio* spp. Associated with Vibriosis incidences in the Greek aquaculture: The role of *Vibrio harveyi* as the principal cause of Vibriosis. Microorganisms.

[2] Lai X., Wu H., Guo W., Li X., Wang J., Duan Y., Zhang P., Huang Z., Li Y., Dong G. (2023). *Vibrio harveyi* co-infected with *Cryptocaryon irritans* to orange-spotted groupers *Epinephelus coioides*. Fish Shellfish Immunol..

[3] Lee Y., Roh H., Kim A., Park J., Lee J.Y., Kim Y.J., Kang H.G., Kim S., Kim H.S., Cha H.J. (2023). Molecular mechanisms underlying the vulnerability of Pacific abalone (Haliotis discus hannai) to *Vibrio harveyi* infection at higher water temperature. Fish Shellfish Immunol..

[4] Zhang X., He X., Austin B. (2020). *Vibrio harveyi*: A serious pathogen of fish and invertebrates in mariculture. Mar. Life Sci. Technol..

[5] Dong H.T., Taengphu S., Sangsuriya P., Charoensapsri W., Phiwsaiya K., Sornwatana T., Khunrae P., Rattanarojpong T., Senapin S. (2017). Recovery of *Vibrio harveyi* from scale drop and muscle necrosis disease in farmed barramundi, *Lates calcarifer* in Vietnam. Aquaculture.

[6] Shen G., Shi C., Fan C., Jia D., Wang S., Xie G., Li G., Mo Z., Huang J. (2017). Isolation, identification and pathogenicity of *Vibrio harveyi*, the causal agent of skin ulcer disease in juvenile hybrid groupers *Epinephelus fuscoguttatus* × *Epinephelus lanceolatus*. J. Fish Dis..

[7] Fan H., Wang L., Wen H., Wang K., Qi X., Li J., He F., Li Y. (2019). Genome-wide identification and characterization of toll-like receptor genes in spotted sea bass (*Lateolabrax maculatus*) and their involvement in the host immune response to *Vibrio harveyi* infection. Fish Shellfish Immunol..

[8] Tian Y., Wen H., Qi X., Mao X., Shi Z., Li J., He F., Yang W., Zhang X., Li Y. (2019). Analysis of apolipoprotein multigene family in spotted sea bass (*Lateolabrax maculatus*) and their expression profiles in response to *Vibrio harveyi* infection. Fish Shellfish Immunol..

[9] Alteri C.J., Mobley H.L.T. (2012). *Escherichia coli* physiology and metabolism dictates adaptation to diverse host microenvironments. Curr. Opin. Microbiol..

[10] Jones S.A., Gibson T., Maltby R.C., Chowdhury F.Z., Stewart V., Cohen P.S., Conway T. (2011). Anaerobic respiration of *Escherichia coli* in the mouse intestine. Infect. Immun..

[11] Jones S.A., Jorgensen M., Chowdhury F.Z., Rodgers R., Hartline J., Leatham M.P., Struve C., Krogfelt K.A., Cohen P.S., Conway T. (2008). Glycogen and maltose utilization by *Escherichia coli* O157:H7 in the mouse intestine. Infect. Immun..

[12] Johnson J.R., Kuskowski M.A., Gajewski A., Soto S., Horcajada J.P., de Anta M.T.J., Vila J. (2005). Extended virulence genotypes and phylogenetic background of *Escherichia coli* isolates from patients with cystitis, pyelonephritis, or prostatitis. J. Infect. Dis..

[13] Johnson J.R., Russo T.A. (2018). Molecular epidemiology of extraintestinal pathogenic *Escherichia coli*. EcoSal Plus.

[14] Russo T., Johnson J.R. (2003). Medical and economic impact of extraintestinal infections due to *Escherichia coli*: Focus on an increasingly important endemic problem. Microbes Infect..

[15] Zhou B., Szymanski C.M., Baylink A. (2023). Bacterial chemotaxis in human diseases. Trends Microbiol..

[16] Yang S., Xi D., Wang X., Li Y., Li Y., Yan J., Cao B. (2020). Vibrio cholerae VC1741 (PsrA) enhances the colonization of the pathogen in infant mice intestines in the presence of the long-chain fatty acid, oleic acid. Microb. Pathog..

[17] Wang H., Xing X., Wang J., Pang B., Liu M., Larios-Valencia J., Liu T., Liu G., Xie S., Hao G. (2018). Hypermutation-induced in vivo oxidative stress resistance enhances *Vibrio cholerae* host adaptation. PLoS Pathog..

[18] Patankar Y.R., Lovewell R.R., Poynter M.E., Jyot J., Kazmierczak B.I., Berwin B. (2013). Flagellar motility is a key determinant of the magnitude of the inflammasome response to *Pseudomonas aeruginosa*. Infect. Immun..

[19] Aso H., Miyoshi S.I., Nakao H., Okamoto K., Yamamoto S. (2002). Induction of an outer membrane protein of 78 kda in *Vibrio vulnificus* cultured in the presence of desferrioxamine B under iron-limiting conditions. Fems Microbiol. Lett..

[20] Lima A., Zunino P., D’Alessandro B., Piccini C. (2007). An iron-regulated outer-membrane protein of *Proteus mirabilis* is a haem receptor that plays an important role in urinary tract infection and in vivo growth. J. Med. Microbiol..

[21] Lan Y., Zhou M., Li X., Liu X., Li J., Liu W. (2022). Preliminary investigation of iron acquisition in hypervirulent *Klebsiella pneumoniae* mediated by outer membrane vesicles. Infect. Drug Resist..

[22] Qin Y., Wang J., Wang S., Yan Q. (2007). Study on the antigenicity of *Vibrio harveyi* TS-628 strain. Front. Biol. China.

[23] Meng X., Shen Y., Wang S., Xu X., Dang Y., Zhang M., Li L., Zhang J., Wang R., Li J. (2019). Complement component 3 (C3): An important role in grass carp (*Ctenopharyngodon idella*) experimentally exposed to *Aeromonas hydrophila*. Fish Shellfish Immunol..

[24] Wang Y., Peng K., Wu J., Chen J. (2014). Transgenic expression of salmon delta-5 and delta-6 desaturase in zebrafish muscle inhibits the growth of *Vibrio alginolyticus* and affects fish immunomodulatory activity. Fish Shellfish Immunol..

[25] Langmead B., Salzberg S.L. (2012). Fast gapped-read alignment with Bowtie. Nat. Methods.

[26] Li B., Dewey C.N. (2011). RSEM: Accurate transcript quantification from RNA-Seq data with or without a reference genome. BMC Bioinform..

[27] Robinson M.D., McCarthy D.J., Smyth G.K. (2010). edgeR: A Bioconductor package for differential expression analysis of digital gene expression data. Bioinformatics.

[28] Chen X., Lin Y., Lu H., Su Y., Liu Z. (2020). Analysis and evaluation of nutritional components in the muscle of four grouper species. J. Fish. Res..

[29] Wang J., Zhang D., Ma J., Li B., Zhang L. (2015). Nutritional components analysis and nutritive value evaluation of *Epinephelus fuscoguttatus* × *Epinephelus lanceolatus* muscles. Trans. Oceanol. Limnol..

[30] Ma Y., Zhang Q., Yang Z., Li Y., Yan Y., Ping S., Zhang L., Lin M., Lu W. (2016). Identification of the nitrogen-fixing *Pseudomonas stutzeri* major flagellar gene regulator FleQ and its role in biofilm formation and root colonization. Agriculture.

[31] Zhang M., Yan Q., Mao L., Wang S., Huang L., Xu X., Qin Y. (2018). KatG plays an important role in *Aeromonas hydrophila* survival in fish macrophages and escape for further infection. Gene.

[32] Jiao J., Zhao L., Huang L., Qin Y., Su Y., Zheng W., Zhang J., Yan Q. (2021). The contributions of *fliG* gene to the pathogenicity of *Pseudomonas plecoglossicida* and pathogen-host interactions with *Epinephelus coioides*. Fish Shellfish Immunol..

[33] Badal D., Jayarani A.V., Kollaran M.A., Prakash D., Monisha P., Singh V. (2021). Foraging signals promote swarming in starving *Pseudomonas aeruginosa*. mBio.

[34] Huang L., Guo L., Xu X., Qin Y., Zhao L., Su Y., Yan Q. (2019). The role of *rpoS* in the regulation of *Vibrio alginolyticus* virulence and the response to diverse stresses. J. Fish Dis..

[35] Rashid M.H., Arthur K. (2000). Inorganic polyphosphate is needed for swimming, swarming, and twitching motilities of *Pseudomonas aeruginosa*. Proc. Natl. Acad. Sci. USA.

[36] Zhang Z., Mao L., Qin Y., Zhao L., Huang L., Xu X., Qin Y. (2022). Comparative transcriptome analysis revealed the role and mechanism of a FeoC-like LuxR-type regulator in intracellular survival of *Aeromonas hydrophila*. Aquaculture.

[37] Wu W., Zhao L., Huang L., Qin Y., Zhang J., Zhao J., Yan Q. (2022). Transcriptomic and metabolomic insights into the role of *fliS* in the pathogenicity of *Pseudomonas plecoglossicida* against *Epinephelus coioides*. Front. Mar. Sci..

[38] Cai H., Ma Y., Qin Y., Zhao L., Yan Q., Huang L. (2023). Vvrr2: A new *Vibrio* ncRNA involved in dynamic synthesis of multiple biofilm matrix exopolusaccharides, biofilm structuring and virulence. Aquaculture.

[39] Wang J., Wu Z., Wang S., Wang X., Zhang D., Wang Q., Lin L., Wang G., Guo Z., Chen Y. (2022). Inhibitory effect of probiotic *Bacillus* spp. isolated from the digestive tract of Rhynchocypris Lagowskii on the adhesion of common pathogenic bacteria in the intestinal model. Microb. Pathog..

[40] Mao L., Qin Y., Kang J., Wu B., Huang L., Wang S., Zhang M., Zhang J., Zhang R., Yan Q. (2019). Role of LuxR-type regulators in fish pathogenic *Aeromonas hydrophila*. J. Fish Dis..

[41] Wooten R.M., Pakkulnan R., Anutrakunchai C., Kanthawong S., Taweechaisupapong S., Chareonsudjai P., Chareonsudjai S. (2019). Extracellular DNA facilitates bacterial adhesion during *Burkholderia pseudomallei* biofilm formation. PLoS ONE.

[42] Qiu X., Cao X., Jian H., Wu H., Xu G., Tang X. (2022). Transcriptomic analysis Reveals that changes in gene expression contribute to *Microbacterium sediminis* YLB-01 adaptation at low temperature under high hydrostatic pressure. Curr. Microbiol..

[43] Cheng Q., McKeown S.J., Santos L., Santiago F.S., Khachigian L.M., Morand E.F., Hickey M.J. (2010). Macrophage migration inhibitory factor increases leukocyte–endothelial interactions in human endothelial cells via promotion of expression of adhesion molecules. J. Immunol..

[44] Kaufmann G.F., Malachowa N., Kobayashi S.D., Sturdevant D.E., Scott D.P., DeLeo F.R. (2015). Insights into the *Staphylococcus aureus*-host interface: Global changes in host and pathogen gene expression in a rabbit skin infection model. PLoS ONE.

[45] Li M., Meng H., Li H., Gu D. (2022). A novel transcription factor VPA0041 was identified to regulate the swarming motility in Vibrio parahaemolyticus. Pathogens.

[46] Dearns D.B. (2010). A field guide to bacterial swarming motility. Nat. Rev. Microbiol..

[47] Miller L.D., Russell M.H., Alexandre G. (2009). Chapter 3 diversity in bacterial chemotactic responses and niche adaptation. Adv. Appl. Microbiol..

[48] Lee S.H., Butler S.M., Camilli A. (2001). Selection for in vivo regulators of bacterial virulence. Proc. Natl. Acad. Sci. USA.

[49] Wang Y., Wu Y., Niu H., Liu Y., Ma Y., Wang X., Li Z., Dong Q. (2023). Different cellular fatty acid pattern and gene expression of planktonic and biofilm state *Listeria monocytogenes* under nutritional stress. Food Res. Int..

[50] Adachi Y., Sousa-Coelho L.D., Harata I., Aoun C., Weimer S., Shi X., Herrera K.N.G., Takahashi H., Doherty C., Noguchi Y. (2018). l-Alanine activates hepatic AMP-activated protein kinase and modulates systemic glucose metabolism. Mol. Metab..

[51] Kim S.H., Schneider B.L., Reitzer L. (2010). Genetics and regulation of the major enzymes of Alanine synthesis in *Escherichia coli*. J. Bacteriol..

[52] Peng B., Su Y., Han Y., Guo C., Tian Y., Peng X. (2015). Exogenous Alanine and/or Glucose plus kanamycin kills antibiotic-resistant bacteria. Cell Metab..

[53] Deng Y., Xie M., Yang Y., Feng J., Tan L., Chen C. (2018). The role of l-Alanine metabolism revealed by transcriptome analysis in *Vibrio alginolyticus*. Gene Rep..

[54] Brijesh K., Cardona S.T. (2016). Synthetic cystic fibrosis sputum medium regulates flagellar biosynthesis through the *flhF* gene in *Burkholderia cenocepacia*. Front. Cell. Infect. Microbiol..

[55] Yang Y., Pollard A.M., Höfler C., Poschet G., Wirtz M., Hell R., Sourjik V. (2015). Relation between chemotaxis and consumption of amino acids in bacteria. Mol. Microbiol..

